# Supplementary material for: The refinement paradox and cumulative cultural evolution: Complex products of collective improvement favor conformist outcomes, blind copying, and hyper-credulity
Source: PLoS Comput Biol. 2024 Sep 26;20(9):e1012436. doi: 10.1371/journal.pcbi.1012436 (PMC11426424; doi:10.1371/journal.pcbi.1012436)
Supplement: S1 Supporting Information — Fig A. Relationship between score and (a) amount of REFINE as a proportion of all learning moves; (b) proportion of just REFINE and INNOVATE learning moves; (c) proportion of INNOVATE moves; (d) proportion of OBSERVE moves and (e) proportion of learning moves that are OBSERVE (and not INNOVATE or REFINE). Fig B. Distribution of final maximum refinement level in the tournament (a) in all simulations, and (b) in simulations that include only entries that use REFINE. Refinement levels above the value of 8 typically lead to payoffs that exceed the maximum basic payoff (Fig 1). The maximum refinement level achieved across simulations varied substantially and showed a bimodal distribution. Fig C. Cultural diversity measures across extensions for Stage I. Cumulative culture leads to decreasing diversity in both the behaviors performed and behaviors known about, as populations converge on a small number of heavily refined, high-payoff behaviors. Fig D. Cultural diversity measures as a function of pc and pcopyFail in Stage 2. Timelines of amount and evenness are presented as line charts, while the boxplots illustrate mean values for mean and maximum persistence. Fig E. Cultural diversity measures as a function of rmax and nObserve, in Stage 2. Fig F. Cultural diversity measures for one run of the cumulative extension in Stage II. (top) Proportion of agents who use and know each act, acts ordered by rank frequency, at increasing timesteps. (bottom) Behaviour and Knowledge evenness over time, with red dots indicating the same timesteps as above. The population very quickly converges on a small set of acts–in the last quarter of the simulation the population knows and uses only one act. Fig G. Amount and evenness of both behavior and knowledge in simulations that only include top ten entries (teal) and simulations with the rest of the 41 entries (red), in Stage I (cumulative extension). Fig H. Mean and maximum persistence of both behavior and knowledge, in simulations that only [file pcbi.1012436.s001.docx]

Supplementary Information for

**The refinement paradox and cumulative cultural evolution:**

**complex products of collective improvement favor conformist outcomes, blind copying, and hyper-credulity**

**Authors:** Elena Miu^1,2,8,11*¶^, Luke Rendell^1*¶^, Sam Bowles^3^, Rob Boyd^2^, Daniel Cownden^4^, Magnus Enquist^5^, Kimmo Eriksson^5^, Marcus W. Feldman^6^, Timothy Lillicrap^7^, Richard McElreath^8^, Stuart Murray^1,9^, James Ounsley^1,10^ & Kevin N. Lala^1*^

Correspondence to: [knl1@st-andrews.ac.uk](mailto:knl1@st-andrews.ac.uk) (KNL), [ler4@st-andrews.ac.uk](mailto:ler4@st-andrews.ac.uk) (LR), [elena.miu@gmail.com](mailto:elena.miu@gmail.com) (EM)

^¶^Joint first authors

**This PDF includes:**

Figs A-I

Tables A-E

Sample entry

**Supplementary Figures**

**
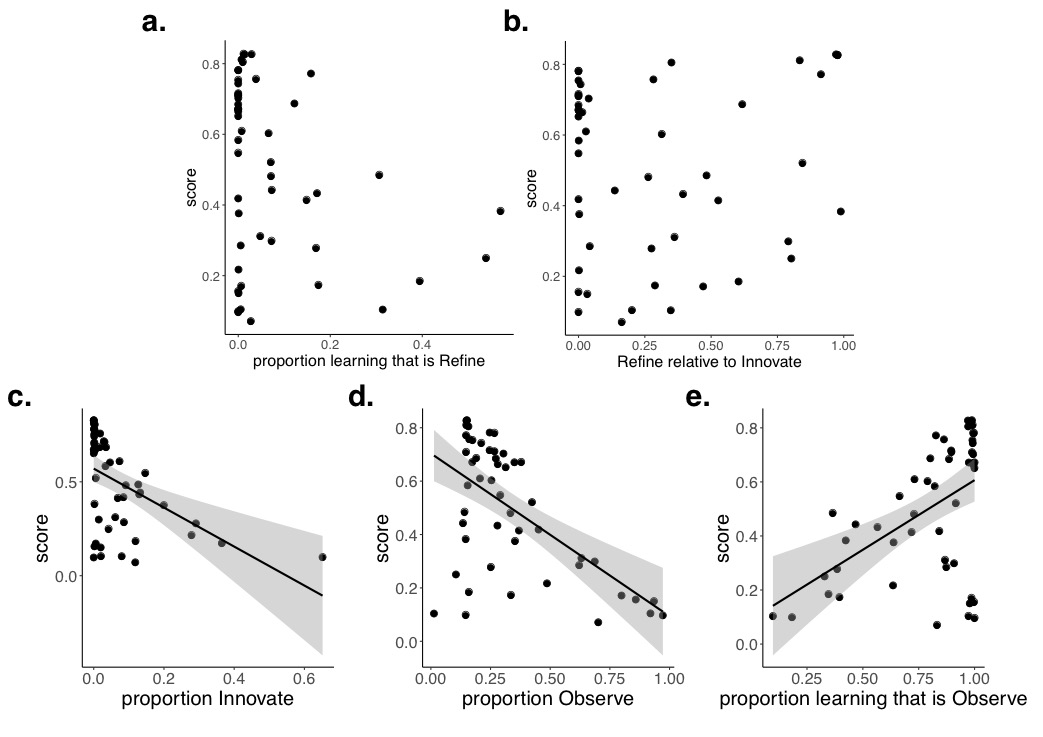
**

Fig A. Relationship between score and (a) amount of REFINE as a proportion of all learning moves; (b) proportion of just REFINE and INNOVATE learning moves; (c) proportion of INNOVATE moves; (d) proportion of OBSERVE moves and (e) proportion of learning moves that are OBSERVE (and not INNOVATE or REFINE).


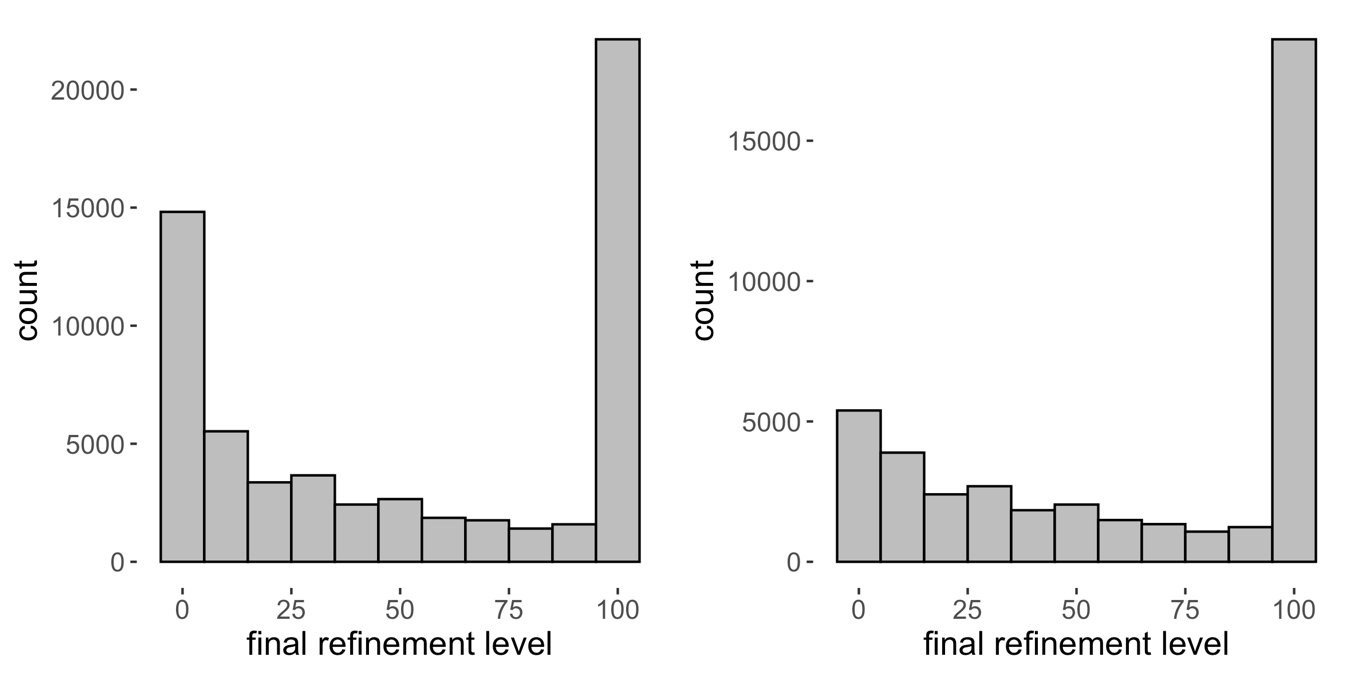


**b.**

**a.**

Fig B. Distribution of final maximum refinement level in the tournament (a) in all simulations, and (b) in simulations that include only entries that use REFINE. Refinement levels above the value of 8 typically lead to payoffs that exceed the maximum basic payoff (Fig. 1). The maximum refinement level achieved across simulations varied substantially and showed a bimodal distribution.

Fig C. Cultural diversity measures across extensions for Stage I. Cumulative culture leads to decreasing diversity in both the behaviors performed and behaviors known about, as populations converge on a small number of heavily refined, high-payoff behaviors.


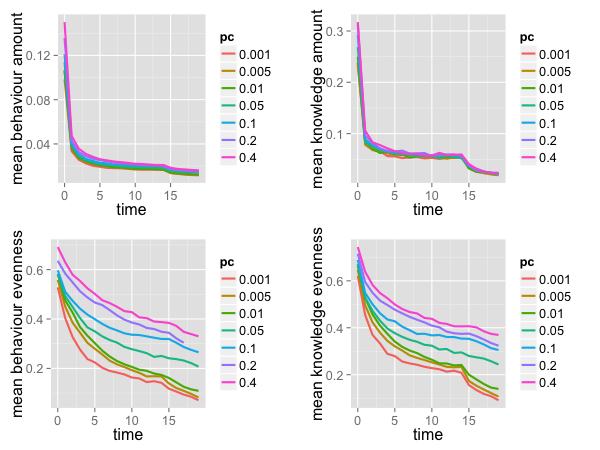

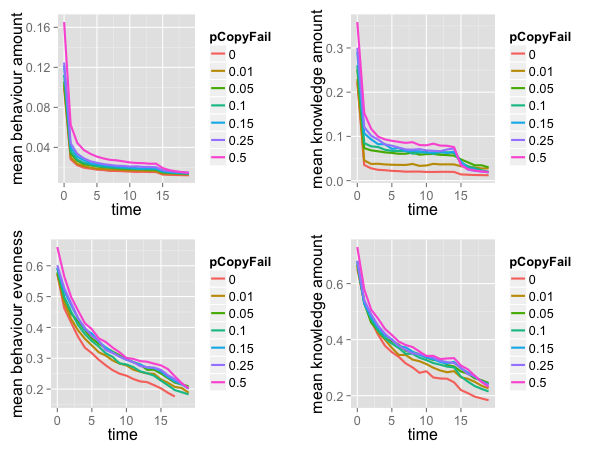

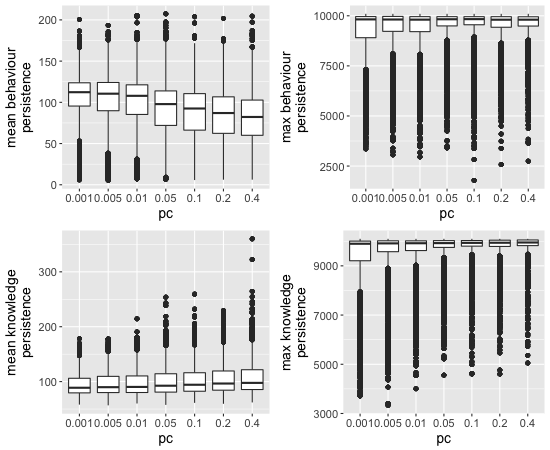

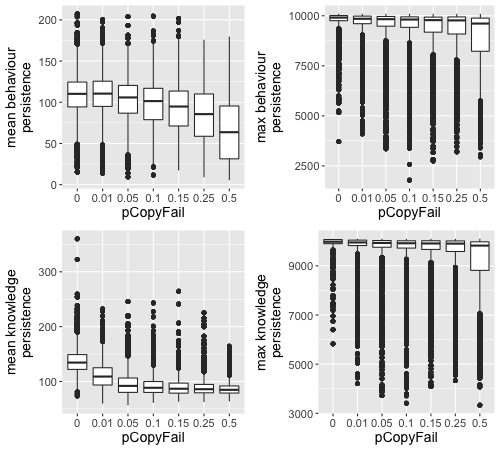


Fig D. Cultural diversity measures as a function of p_c_ and p_copyFail_ in Stage 2. Timelines of amount and evenness are presented as line charts, while the boxplots illustrate mean values for mean and maximum persistence.


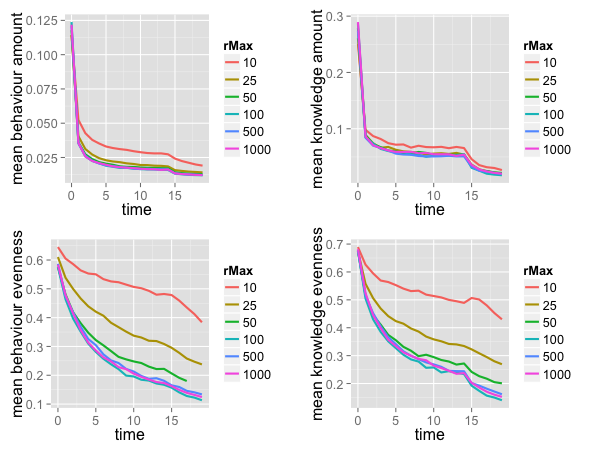

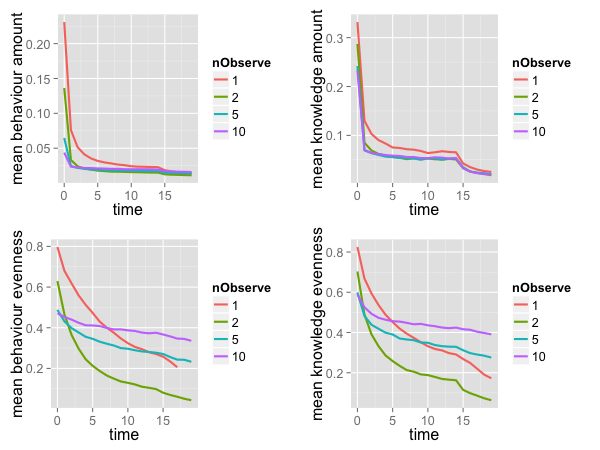

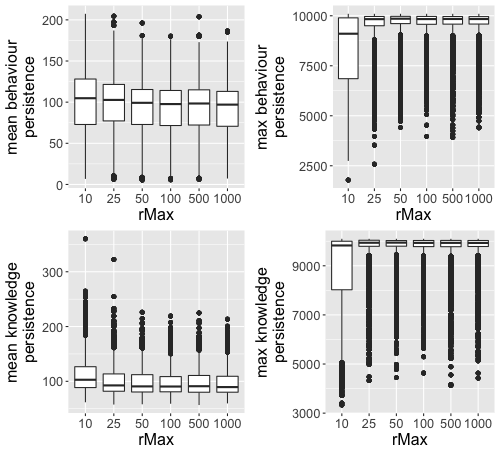

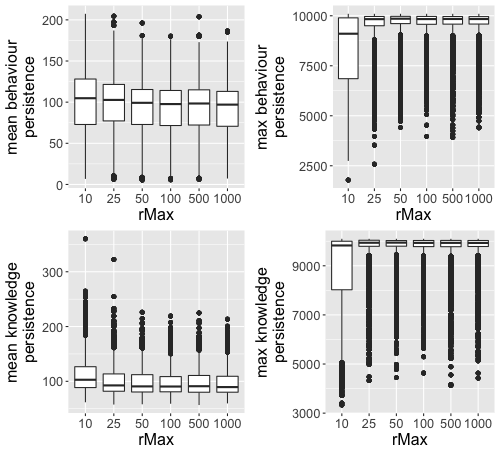


Fig E. Cultural diversity measures as a function of r_max_ and n_Observe_, in Stage 2.

Fig F. Cultural diversity measures for one run of the cumulative extension in Stage II. (top) Proportion of agents who use and know each act, acts ordered by rank frequency, at increasing timesteps. (bottom) Behaviour and Knowledge evenness over time, with red dots indicating the same timesteps as above. The population very quickly converges on a small set of acts – in the last quarter of the simulation the population knows and uses only one act.


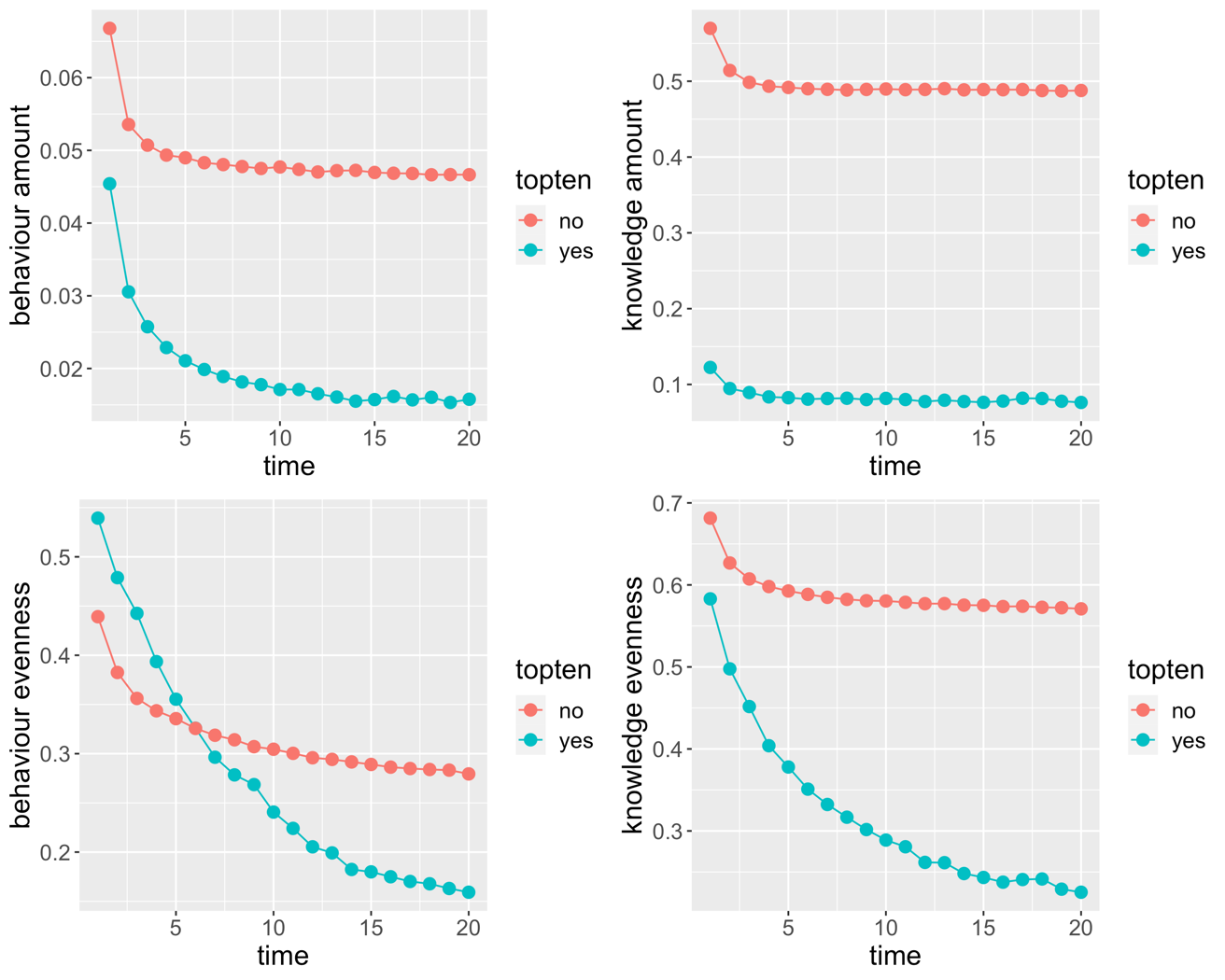


Fig G. Amount and evenness of both behavior and knowledge in simulations that only include top ten entries (teal) and simulations with the rest of the 41 entries (red), in Stage I (cumulative extension).

Fig H. Mean and maximum persistence of both behavior and knowledge, in simulations that only include the top ten entries (teal) and simulations with the rest of the 41 entries (red), in Stage I (cumulative extension).


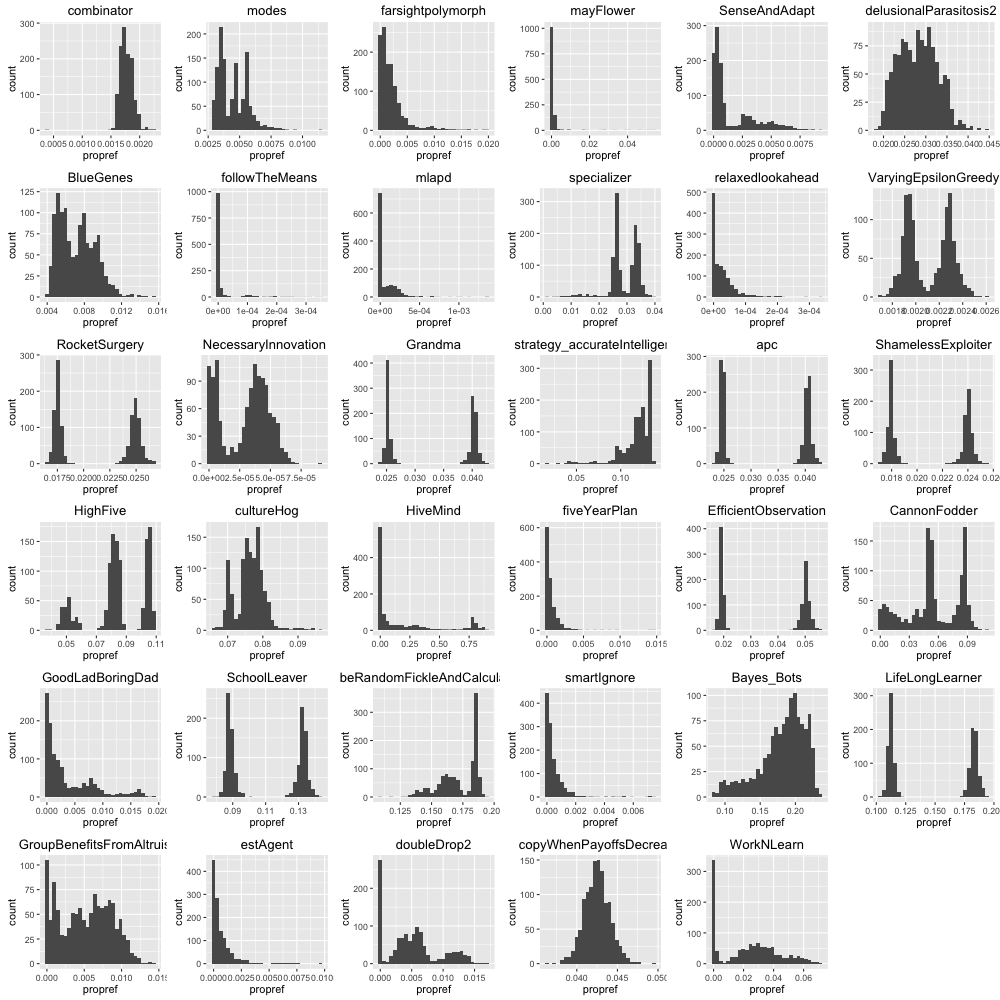


Fig I. Distribution of mean proportion REFINE moves per simulation for each entry, ordered from the top left by score in Stage 1, in descending order. This plot only includes the 35 entries that used the REFINE move. Note the difference in scale on the x axis. The top four entries were also the top performers in Stage 3, which establishes that the best-performing entry used REFINE at low levels.

**Supplementary Tables**

Table A. Model averaged parameter estimates and summed Akaike weights (across an all-subsets model set of linear regression models with Gaussian error) for Stage 1 cumulative extension. Other extensions give similar findings (see Table S2). The AIC_c_ best model (adjusted R-squared=0.86) contained *pLearnRefine* and *pObserve*, as well as *meanPayDiffObserve*, *meanBetweenLearn* and *doRefine*. The first two predictors were included in all of the 13 well-supported (ΔAIC_c_<2) models; while the others were in 9, 6 and 7 respectively.

| **Term** | **Definition** | **Estimate** | **s.e** | **∑ω*_i_*** |
| --- | --- | --- | --- | --- |
| pLearnRefine | Proportion of all moves that were either REFINE, INNOVATE, or OBSERVE i.e. not EXPLOIT. | -0.798 | 0.078 | 1.000 |
| pObserve | Proportion of learning moves (OBSERVE +INNOVATE) that were OBSERVE. | 0.379 | 0.077 | 1.000 |
| doRefine | Does the entry play, or have the possibility of playing the REFINE move? (scored 0/1) | -0.057 | 0.035 | 0.529 |
| meanPayDiffObserve | The average difference in payoffs between EXPLOIT moves immediately preceding an OBSERVE move and the next EXPLOIT move after it. | -0.001 | 0.001 | 0.521 |
| meanBetweenLearn | The average number of rounds between learning moves. | -0.004 | 0.003 | 0.406 |
| pFailEst | Does the entry estimate *pcopyFail*, the probability that observing fails? (scored 0/1). | -0.070 | 0.058 | 0.354 |
| checkPayDist | The entry uses some aspect of the agent’s historical payoffs in deciding how to move – either the maximum or mean of all the payoffs in the history, or possibly some subset of the entire history. | -0.036 | 0.035 | 0.309 |
| payDrop | Does the entry have a specific rule for dealing with the situation in which the payoff for a known behaviour drops below some threshold after being used in an EXPLOIT move? (scored 0/1) | -0.030 | 0.032 | 0.295 |
| pcEst | Does the entry estimate *p_c_*, the probability of environmental change? (scored 0/1) | 0.032 | 0.036 | 0.276 |
| lineLength | The number of lines in the Python source code for the entry. | <0.001 | <0.001 | 0.257 |
| checkRefInc | Does the entry calculate the payoff increments before and after REFINE has been played using a particular behaviour? (scored 0/1) | 0.029 | 0.040 | 0.249 |
| discountsOld | Does the entry perform some sort of discounting of historical information, either by truncating histories or weighting older data using some function of the age of the information? (scored 0/1) | 0.020 | 0.041 | 0.226 |
| nObsEst | Does the entry estimate the number of other agents surveyed in an OBSERVE move, the simulation parameter *nobserve*? (scored 0/1) | 0.010 | 0.036 | 0.206 |
| poly | An entry is described as polymorphic if the agent deploying it may be of more than one “type” (e.g., sometimes producer and sometimes scrounger). For instance, some entries feature a random choice made at birth and signalled in the history by a particular move, which then defines their behaviour. In this way, a population of agents may be split into different groups, each playing different entries. | 0.007 | 0.041 | 0.200 |
| meanRoundsToExploit | The average number of rounds between the birth of an agent with this entry and that agent’s first EXPLOIT move | 0.001 | 0.003 | 0.199 |

Table B. Results from a linear mixed model predicting score as a function of the interaction between whether an environment was refined or not and whether an entry used refine or not, with a varying intercept for entry identity, using data for all 51 entries in the tournament. The model definition: score ~ environment type*entry type + 1|entry, assuming a non-refine entry in a non-refined environment as baseline.

| Variable | β coefficient | Standard error | t-value |
| --- | --- | --- | --- |
| Intercept | 0.568 | 0.064 | 8.851 |
| refined environment | 0.010 | 0.006 | 1.616 |
| refine entry | -0.175 | 0.077 | -2.261 |
| refined environment * refine entry | 0.161 | 0.007 | 22.115 |

Table C. Results from a similar linear mixed model with a varying intercept for entry identity, using data for 20 entries, the top-scoring 10 entries that used REFINE and the top-scoring 10 entries that did not use REFINE. Model definition: score ~ environment type*entry type + 1|entry, assuming a non-refined entry in a non-refined environment as baseline.

| Variable | β coefficient | Standard error | t-value |
| --- | --- | --- | --- |
| Intercept | 0.706 | 0.017 | 40.891 |
| refined environment | 0.068 | 0.007 | 8.913 |
| refine entry | 0.044 | 0.024 | 1.826 |
| refined environment * refine entry | 0.029 | 0.020 | 2.890 |

Table D. Definitions of new terms

| **Term** | **Definition** |
| --- | --- |
| Learning move | Any move used to acquire new behavior or revise present behavior, i.e. all moves but EXPLOIT |
| Refine entry | Entries that make use of the REFINE move |
| Refined environment | Environments in which the refinement level of at least one behavior has reached the maximum level, 100 |
| ‘Clever’ refiner | Comprised the 6 entries that were placed in the top 10, consistently played REFINE, and used REFINE in a strategic manner. |

Table E. Simulation parameters

*Stage I*

*Single extension pairwise*

| **Parameter** | **Values** |
| --- | --- |
| *pc* | {0.001, 0.01, 0.1} |
| *pcopyFail* | 0.05 |
| *nobserve* | {1, 5} |
| *rmax* | 100 |

*Stage II*

*Single extension melee*

| **Parameter** | **Values** |
| --- | --- |
| *pc* | {0.001, 0.005, 0.01, 0.05, 0.1, 0.2, 0.4} |
| *pcopyFail* | {0, 0.01, 0.05, 0.1, 0.15, 0.25, 0.5} |
| *nobserve* | {1, 2, 5, 10} |
| *rmax* | {10, 25, 50, 100, 500, 1000} |

*Stage II*

*All extension melee*

| **Parameter** | **Values** |
| --- | --- |
| *pc* | {0.001, 0.005, 0.01, 0.05, 0.1, 0.2, 0.4} |
| *pcopyFail* | {0, 0.01, 0.05, 0.1, 0.15, 0.25, 0.5} |
| *nobserve* | {1, 2, 5, 10} |
| *rmax* | {10, 25, 50, 100, 500, 1000} |

Sample entry – winning entry farSightPolymorph

import numpy as np

import numpy

from moves import * # bring in standard names for moves

import random,math

def move(roundsAlive, repertoire, historyRounds, historyMoves, historyActs, historyPayoffs, historyDemes, currentDeme, canChooseModel, canPlayRefine, multipleDemes):

# Put the history into convient numpy arrays.

hacts = np.asarray(historyActs)

hdemes = np.asarray(historyDemes)

hmoves = np.asarray(historyMoves)

hpayoffs = np.asarray(historyPayoffs)

hrounds = np.asarray(historyRounds)

hist = np.asarray([hrounds, hmoves, hacts, hpayoffs, hdemes])

if roundsAlive == 0:

# Try to observe on the first round

return (OBSERVE, )

elif len(repertoire) == 0:

# If you still don't know anything, try to observe some more

if roundsAlive > 15:

# After 15 fruitless observes in a row it is time to innovate.

# This happens during the burn in.

# When probability of observation failling is 0.5, and the number

# of observations per move is 1 there is a less than a 1/20000

# chance of this happening, which means less than once a simulation

# on average.

return (INNOVATE, )

return (OBSERVE, )

else:

# Now we actually run our creature

# Good to know how old you are

t = roundsAlive

# and the probability of death

psubd = 0.02

# Sometimes we would like to take a large sum, if there are more than trunc terms in that sum, we settle

# for approximating that sum by the sum of the first trunc terms

trunc = 50

#We crawl through the critter's repertoire, simultaneously estimating psubc, and two quantities related to

#the mean of the payoff distribution. One called allmeanpayoff and the other called trimmeanpayoff.

#The estimate allmeanpayoff will be biased up because it includes acts that have been learned through observation

#which will often be of a higher payoff than a randomly selected act. The estimate trimmeanpayoff on the other hand will

#only be biased up slightly, and only in the refine case, because new acts are often learned through observations, and

#those observed acts may be refined.

#The estimate of psubc is imperfect because it does not take into account information from refine moves, nor information

#from non-consecutive rounds. (This can cause a flip flop not data issue which we need to address)

#allmeanpayoff will be the average of all of the distinct payoffs preceived. In contrast, trimmeanpayoff, will be the

#average of all of the distinct payoffs preceived, excluding those directly resulting from an observation.

alldistinctpayoffs = np.array([],np.float32)

trimdistinctpayoffs = np.array([],np.float32)

# psubc will be estimated by dividing the expected number of odserved act payoff changes

# divided by the number of opportunities for such change.

psubcnumerator = 0

psubcdenomenator = 0

# secondpasspsubc will be used to store information about act payoff changes that are non

# consecutive, which will be incorporated into the psubc estimate after all the other

# information has been used to create a first pass estimate of psubc

secondpasspsubc = []

for ii in repertoire.iterkeys():

# Look at all the places where you have exploited, refined or innovated

# the act, ii, in question, and hence know its payoff with certainty

expii = (hacts==ii) & ((hmoves == EXPLOIT) | (hmoves == INNOVATE) | (hmoves == REFINE))

if (expii.sum() > 1.):

#Find the places where the quality of ii is known with certainty, and count the number

#of times this payoff changes. Note that we are not considering refined or observed acts here.

paydifferences = np.diff(hpayoffs[expii])

timedifferences = np.diff(hrounds[expii])

demedifferences = np.diff(hdemes[expii])

consecutive = (timedifferences==1)

paychange = ~(paydifferences==0)

demechange = ~(demedifferences==0)

# psubc is then estimated as the number of times when there has been a measured change in the quality of a given act,

# from one round to the next, divided by the total number of times that that the payoff resulting from a given act is

# known with certainty

psubcdenomenator = psubcdenomenator + (consecutive & ~demechange).sum()

psubcnumerator = psubcnumerator + (consecutive & ~demechange & paychange).sum()

# If the same act is observed to be at the same value (provided that value is strictly greater than 4) then

# if the number of intervening rounds is less than 6 we assume that it has not changed in that time and so

# adjust psubcdenomenator accordingly.

psubcdenomenator = psubcdenomenator + (timedifferences[ (~paychange) &

(~demechange) &

(timedifferences < 6) &

((hpayoffs[expii] > 6)[0:-1]) &

(~consecutive)]).sum()

secondpasspsubc.append(timedifferences[(paychange) & (~demechange) & (~consecutive)])

# meanpayoff is then estimated as the mean of each distinct, exploited payoff for each act.

# Note that payoffs observed during the refine act are not used in this estimate, however

# if the payoff of an act has changed soley because the critter has refined it, this new

# refined payoff for the act is treated as distinct for purposes of estimating the mean

# while this is not technically correct, it is computationally simpler.

distinctpayoffsii = hpayoffs[expii][np.hstack(([True], (paychange | demechange)))]

if (hmoves[hacts==ii][0] == OBSERVE):

alldistinctpayoffs = np.hstack((alldistinctpayoffs, distinctpayoffsii))

trimdistinctpayoffs = np.hstack((trimdistinctpayoffs, distinctpayoffsii[1:]))

elif(hmoves[hacts==ii][0] == INNOVATE):

alldistinctpayoffs = np.hstack((alldistinctpayoffs, distinctpayoffsii))

trimdistinctpayoffs = np.hstack((trimdistinctpayoffs, distinctpayoffsii))

elif (expii.sum() == 1.):

distinctpayoffsii = hpayoffs[expii]

if (hmoves[hacts==ii][0] == OBSERVE):

alldistinctpayoffs = np.hstack((alldistinctpayoffs, distinctpayoffsii))

#only have the observed value of this don't use it in trimdistinctpayoffs

elif(hmoves[hacts==ii][0] == INNOVATE):

alldistinctpayoffs = np.hstack((alldistinctpayoffs, distinctpayoffsii))

trimdistinctpayoffs = np.hstack((trimdistinctpayoffs, distinctpayoffsii))

#else:

#This act has only been observed. These observations (that's right there may be more than one)

#will get incorporated into the estimate of the mean of the payoff distribution a little later.

# Even though we are not done estimating the meanpayoff, we will need a first pass estimate

# of meanpayoff when computing qobs.

if len(alldistinctpayoffs) == 0:

# If you've only ever observed this will happen. it shouldn't get used, but is a safe gaurd.

tmpobsmeanpayoff = 2.

else:

tmpobsmeanpayoff = alldistinctpayoffs.mean()

# Now we incorporate information from observations followed by exploits

# into the estimate of psubc. While we're at it we estimate qobs.

# We first note all the rounds on which there is an observe. Each of these

# observe moves becomes a data point for estimating the quality of the actions

# discovered through observation.

observerounds = set(hrounds[hmoves==OBSERVE])

obsdata = []

# Go through each round where there is an observe

for rr in observerounds:

currindx = (hrounds==rr)

nextindx = (hrounds>rr)

nextmoves = hmoves[nextindx]

observedacts = hacts[currindx]

nextacts = hacts[nextindx]

nextpayoffs = hpayoffs[nextindx]

nextdemes = hdemes[nextindx]

observeddeme = hdemes[currindx][0]

just_use_obs = False

if (nextmoves.size == 0):

# If this observe is the last thing you have done

just_use_obs = True

elif ((nextmoves[0] == EXPLOIT) & (nextdemes[0] == observeddeme) & ((nextacts[0] == observedacts).any())):

# If this observe is followed by an exploit, at the same deme.

expbag = []

for ii in range(min(5,nextmoves.size)):

#Find the exploits following the observe

# Are we exploiting one of the actions that we just observed, and are we still at the same deme

if ((nextmoves[ii] == EXPLOIT) & (nextdemes[ii] == observeddeme) & ((nextacts[ii] == observedacts).any())):

#If this is not our first time exploiting after that observe

if (ii > 0):

#Are we still looking for an act or have we settled in.

if (nextacts[ii]==nextacts[ii-1]):

#If we have settled into an action don't add anything more to expbag

break;

# If you haven't settled in yet add another datapoint to the expbag.

#The payoff of the act you just exploited is expval

expval = np.float64(nextpayoffs[ii])

#The mean of the observations associated with the act you just exploited is obsval

tmp = (hacts == nextacts[ii]) & (hrounds == rr)

obsval = np.float64(hpayoffs[tmp].mean())

#We use the fact that poisson noise is approximately

#gaussian to compute a confidence intervals

lowbound = expval - 0.5 - np.sqrt(expval) * 2.57

midlowbound = expval - 0.5 - np.sqrt(expval) * 0.06

midupperbound = expval - 0.5 + np.sqrt(expval) * 0.06

upperbound = expval - 0.5 + np.sqrt(expval) * 2.57

if obsval > upperbound:

#The obsval payoff is so much greater than the expval payoff that we

#are almost certain that that payoff has changed between when the act

#was observed and when it was exploited (the critter is wrong in this way

#only 1 in 200 times.

expbag.append(obsval)

alldistinctpayoffs = np.hstack((alldistinctpayoffs, np.asarray(obsval)))

trimdistinctpayoffs = np.hstack((trimdistinctpayoffs, np.asarray(expval)))

if ii == 0:

psubcdenomenator = psubcdenomenator + 1

psubcnumerator = psubcnumerator + 1

elif obsval > midupperbound:

#Not so sure wheather there was a change between observing or exploiting,

#be safe and use the expval payoff for computing qobs

expbag.append(expval)

elif obsval > midlowbound:

#Almost certain that the payoff has not changed between when the act was observed

#and when it was exploited. expval is thrown into the expbag for computing qobs,

#and this data is used in estimating psubc, allmeanpayoff,

expbag.append(obsval)

if ii == 0:

psubcdenomenator = psubcdenomenator + 1

elif obsval > lowbound:

#Not so sure wheather there was a change between observing or exploiting,

#be safe and use the expval payoff for computing qobs

expbag.append(expval)

else:

#The obsval payoff is so much less than the expval payoff that we

#are almost certain that that payoff has changed between when the act

#was observed and when it was exploited (the critter is wrong in this way

#only 1 in 200 times.

expbag.append(obsval)

alldistinctpayoffs = np.hstack((alldistinctpayoffs, np.asarray(obsval)))

trimdistinctpayoffs = np.hstack((trimdistinctpayoffs, np.asarray(expval)))

if ii == 0:

psubcdenomenator = psubcdenomenator + 1

psubcnumerator = psubcnumerator + 1

else:

#If we are not exploiting one of the actions that we just observed, break out.

break

expbag = np.asarray(expbag)

#use the mean and the max of the exploits in the expbag to estimate the value of qobs

obsdata.append((expbag.mean() + expbag.max())/2.)

else:

# if this observe is not followed by and exploit treat just like

# the case where the observe move was the last move to have been made

just_use_obs = True

# If for whatever reason there are no exploits corresponding to this

# observation move we simply use the observed payoff for our estimate of qobs

if just_use_obs:

obspayoffs = hpayoffs[currindx]

obsacts = hacts[currindx]

obsacts = obsacts[obspayoffs > -1]

obspayoffs = obspayoffs[obspayoffs > -1]

if (obspayoffs.size == 0):

#If all your observes failed

pass

else:

#Use the mean and the max of these observed values to approximate qobs

# Average the payoffs from the same actions

uniqueacts = set(obsacts)

newobspayoffs = np.zeros(len(uniqueacts))

sameobscount = 0;

for aa in uniqueacts:

obsval = obspayoffs[obsacts==aa].mean()

newobspayoffs[sameobscount] = obsval

sameobscount = sameobscount + 1

# In addition we use this as a datapoint for estimating allmeanpayoff,

# though not trimmeanpayoff. This is risky in some cricumstances.

# When the population is exploiting a relatively small number of acts,

# and the same acts are observed over and over again, a bias will be

# introduced. We hope that in this case the critter will learn to not

# observe so much and so minimize this issue.

alldistinctpayoffs = np.hstack((alldistinctpayoffs, np.asarray(obsval)))

obsdata.append((newobspayoffs.mean() + newobspayoffs.max())/2.)

# After we've gone through each act in the repertoire, and incorporated information from

# observations when appropriate we compute our estimate of psubc and the meanpayoff.

if (psubcdenomenator < 4):

# If we've only got a few data points extreme and inaccurate estimates are likely,

# thus we assume a middling value of as a default until we have at least 4 data points

psubc = 0.05

else:

tmppsubc = np.float32(psubcnumerator) / np.float32(psubcdenomenator)

#If this estimate is too high or too low shift it to the known maximal or minal bounds on psubc

if (tmppsubc > 0.4):

tmppsubc = 0.4

elif (tmppsubc < 0.001):

lb = 0.001

n = psubcdenomenator

tmpindx = np.arange(1.,n+1.)

tmppsubc = lb * (1./(1.-lb))**(n+1.) *( - np.log(lb) - ((1.-lb**tmpindx) / tmpindx).sum() )

#Using this estimate of psubc we can now use the data from non consecutive exploits of the same act,

#where the payoff has changed at least once in the td rounds between being known with certainty

newpsubcdenomenator = psubcdenomenator

newpsubcnumerator = psubcnumerator

for timedifs in secondpasspsubc:

#When we went through the repertoire originally we set aside the time difference for each act

#where the payoff had changed, but the deme had not, and set them aside in secondpasspsubc.

#In this case we know that there has been a least one change in the time interval, and we

#use our estimate of psubc thus far to estimate the number of additional changed

newpsubcdenomenator = newpsubcdenomenator + timedifs.sum()

newpsubcnumerator = newpsubcnumerator + len(timedifs) + (timedifs.sum() - len(timedifs))*tmppsubc

psubc = np.float32(newpsubcnumerator) / np.float32(newpsubcdenomenator)

if (psubc > 0.4):

psubc = 0.4

elif (psubc < 0.001):

lb = 0.001

n = newpsubcdenomenator

tmpindx = np.arange(1.,n+1.)

psubc = lb * (1./(1.-lb))**(n+1.) *( - np.log(lb) - ((1.-lb**tmpindx) / tmpindx).sum() )

# be more conservative:

psubc = (tmppsubc + psubc)/2.

#Compute our estimate of allmeanpayoff

if (len(alldistinctpayoffs) == 0):

#If we have yet to exploit or innovate, use the mean of everything to compute allmeanpayoff estimate

allmeanpayoff = (hpayoffs[hpayoffs > 0]).mean()

#This could potentially be nan, if we have also only ever observed things with a zero value, or failed to observe

#In this case set allmeanpayoff at the extremely low value of 2.

if np.isnan(allmeanpayoff):

allmeanpayoff = 2

else:

#Usually though we will have data from which to make a sensible estimate

allmeanpayoff = alldistinctpayoffs.mean()

#Compute our estimate of trimmeanpayoff

if (len(trimdistinctpayoffs)) == 0:

#If we have yet to exploit something that has changed from it's observed value, or to innovate an act, use

#allmeanpayoff as trimmeanpayoff.

trimmeanpayoff = allmeanpayoff - 1.

else:

trimmeanpayoff = np.asarray(trimdistinctpayoffs,np.float32).mean()

qinv = trimmeanpayoff

# We would like an estimate of the the probability of migrating to another deme, psubm

if multipleDemes:

if roundsAlive > 3:

#number of times you're deme has changed

psubmnumerator = (~(np.diff(hdemes) == 0)).sum()

#number of rounds it could have changed

psubmdenominator = hrounds[-1]-1

psubm = np.float32(psubmnumerator) / np.float32(psubmdenominator)

#If psubm is outside of it's known range push it back in there.

if psubm < 0.01:

psubm = 0.01

elif psubm > 0.05:

psubm = 0.05

else:

psubm = 0.02

else:

psubm = 0

#Now we crawl through the repertoire again, this time estimating the quality of each act in the repertoire

#and if possible the refinement level of each act.

for ii in repertoire.iterkeys():

actdemes = hdemes[hacts==ii]

thisdeme = (actdemes==currentDeme)

actmoves = hmoves[hacts==ii]

actmoves_curdeme = actmoves[thisdeme]

actrounds = hrounds[hacts==ii]

actrounds_curdeme = actrounds[thisdeme]

actpayoffs = hpayoffs[hacts==ii]

actpayoffs_curdeme = actpayoffs[thisdeme]

#If you have some experience of this act at your current deme

if len(actpayoffs_curdeme) > 0:

acttimediffs = actrounds_curdeme[-1] - actrounds_curdeme

lastmove = actmoves_curdeme[-1]

timesincelast = hrounds[-1] - actrounds_curdeme[-1]

if (lastmove == OBSERVE):

# If the most recent information we have about this act is from an observation

# and hence uncertain we want to incorporate information from earlier preceptions

# of the payoff of this act.

recjj = 0

# look back in time until you hit something solid (i.e. exp/ref/inv or until the beginning:

for jj in range(len(actmoves_curdeme)-1,-1,-1):

if ~(actmoves_curdeme[jj] == OBSERVE):

recjj = jj

break

T = (1.-psubc)**acttimediffs[recjj:]

Tdenomenator = T.sum()

lastpayoff = sum(actpayoffs_curdeme[recjj:] * T)/Tdenomenator

else:

lastpayoff = actpayoffs_curdeme[-1]

# The expected value of the act goes into the repertoire

repertoire[ii] = ( (1.-(1.-psubc)**(timesincelast+1)) * trimmeanpayoff

+ ((1.-psubc)**(timesincelast+1)) * lastpayoff )

else:

# If you have no experience of this act at your current deme

# The expected value is slotted into the repertoire.

repertoire[ii] = trimmeanpayoff

bestact = max(repertoire, key=repertoire.get)

qexp = repertoire[bestact]

if not(canPlayRefine):

# Only in the non refine cases are we actually interested in the value of observation.

# Here is where we estimate all the little parameters that are helpful when determining

# whether or not to observe

#"Estimate" nobserve:

nobserve = (hrounds==1).sum()

#Estimate psubf, the probability that observation fails

tmp = (hmoves == OBSERVE)

numtrials = tmp.sum()

numfails = (hacts[tmp] == -1).sum()

if (numtrials == 0):

psubf = 0

else:

psubf = np.float32(numfails)/np.float32(numtrials)

# If we estimate psubf to be higher than the allowed range, clip it.

if (psubf > 0.5):

psubf = 0.5

del(tmp,numtrials,numfails)

# now we want to calculate the chances of all observes failing on a given round.

totpsubf = psubf**(np.float32(nobserve))

# We want use the data from the more recent observes more prominently than from older observes

# because the distribution of the quality of observable acts will in general not be stable

# over time, either because there is a small number of distinct acts in the cultural repertoire,

# and so the chance fluctuation of the quality of this small number of acts will change the sampling

# distribution for qobs, or because refinement is causing an upward trend in the qobs distribution.

# either way older data should not count for as much as new data.

if not(obsdata):

qobs = tmpobsmeanpayoff + 1.

else:

wackyweights = 0.95**(np.arange(0,len(obsdata)))

wackyweights[0] = 1.

wackyweights = wackyweights[::-1]

qobs = np.sum(wackyweights*np.array(obsdata))/wackyweights.sum()

#If we only have one observe then use everythin we've got to estimate qobs

if (len(observerounds)==1):

qobs = alldistinctpayoffs.max()

#Often in the simulation it will be the case that only a very small number of distinct actions are being exploited

#by the population at any given moment in time, and further that the population repertoire of known actions is also

#very small. In this case a critter that already knows most of the acts that are currently being employed by the

#population, will likely do better to exploit or refine one of these acts, or innovate a new act. Thus if a critter

#has observed an act that it already knows then it will not consider the observe move, but only the innovate, exploit

#and refine moves for obs_cooldown rounds. To achieve this behaviour the critter forgoes observing in either of the

#following two cases.

#1: The ratio of observations made to acts in the repertoire falls below a critical threshold.

#2: The forager has observed within some specified cool down period, and when they observed they learned no new act.

obs_stale = False

stalethreshold = 0.25

numobservations = len(obsdata)

observedacts = hacts[(hmoves==OBSERVE)]

distinctobservedacts = set(observedacts)

distinctobservedacts.discard(-1)

#Final stale mate:

if numobservations > 6:

stale_obs_estimate = np.float32(len(distinctobservedacts)) / np.float32(numobservations)

if stale_obs_estimate < stalethreshold:

obs_stale = True

if ((roundsAlive > 4) & (not((currentDeme != hdemes[-4:-1]).any()))):

if not obs_stale:

obs_cooldown = 4

#if there has ever been at least 3 observes

if numobservations > 1:

lastobserveround = hrounds[(hmoves==OBSERVE) & (hacts > -1)][-1]

#and if the most recent observe was in the last obs_cooldown rounds

if lastobserveround > (roundsAlive - obs_cooldown):

recentlyobservedacts = set(hacts[(hrounds==lastobserveround)])

recentlyobservedacts.discard(-1)

#and if every recently observed act was already known

if recentlyobservedacts.issubset(hacts[hrounds < lastobserveround]):

#then observation is considered stale and will not be done

obs_stale = True

#This is where we do all the decision making for the non-REFINE cases

# We are interested in the expectation of several quantities which are function of the random variable, m,

# which denotes the number of rounds that critter will have exploiting a given act before a migration or

# environmental change event alters the quality of a given act for a critter.

# Ideally we would like to compute the expected value of these function with respect to the distribution of m,

# however this is too computationally costly given the tournament restrictions. Thus we must content ourselves

# with considering the two integer values on either side of the expected value of m, denoted mf and mc for

# floor and cieling respectively. We then evaluate the various functions of m that we are interested in at

# these two points. We then treat a wieghted average of these two evaluations as a good proxy E[f(m)].

# Thus even though it is not true that E[f(m)] = f(E[m]), we act as though it is and use the

# approximation E[f(m)] = mfw * f(mf) + mcw * f(mc).

if multipleDemes:

mtmp = (1-psubc)*(1-psubm) / (1 - (1-psubc)*(1-psubm))

else:

mtmp = (1-psubc)/psubc

mf = numpy.floor(mtmp)

mc = numpy.ceil(mtmp)

if (mf == mc):

mfw = 0.5; mcw = 0.5

else:

mfw = mc-mtmp

mcw = mtmp-mf

# deltamf and deltamc are approximations of how the rewards recieved from an act continue to benefit the

# critter's fitness over the course of it's life after the rewards have been recieved for m rounds

deltamf = ( ((1-psubd)**(np.arange(mf+1.,mf+1.+trunc))) / (np.arange(t+mf,t+mf+trunc)) ).sum()

deltamc = ( ((1-psubd)**(np.arange(mc+1.,mc+1.+trunc))) / (np.arange(t+mc,t+mc+trunc)) ).sum()

# var_now_mf and var_now_mc compute how the rewards recieved from an act contribute to the critter's

# fitness over the m rounds that they are received, given that there is no delay in the reciept of

# these rewards, since the critter is exploiting an act that it is already aware of, and so need not

# waste a round observing, refining, or innonvating.

# var_now_mf = ((np.arange(1.,mf+1.) / np.arange(t,t+mf)) * ((1-psubd)**(np.arange(1.,mf+1.)))).sum()

# var_now_mc = ((np.arange(1.,mc+1.) / np.arange(t,t+mc)) * ((1-psubd)**(np.arange(1.,mc+1.)))).sum()

# var_delay_mf and var_delay_mc compute how the rewards recieved from an act contribute to the critter's

# fitness over the m rounds that they are received, given that there is a one round delay in the reciept

# of these rewards, since the critter first had to observe, refine, or innovate the act that it will be

# exploiting for these m rounds

# var_delay_mf = ((np.arange(1.,mf) / np.arange(t+1.,t+mf)) * ((1-psubd)**(np.arange(2.,mf+1.)))).sum()

# var_delay_mc = ((np.arange(1.,mc) / np.arange(t+1.,t+mc)) * ((1-psubd)**(np.arange(2.,mc+1.)))).sum()

# What follows is a more computationally efficeint way of computing var_delay, var_now, and also the

# the extension to the case where there is a delay of longer than one round in learning a payoff

# Thus var_now = d1, var_delay = d2, etc.

a = np.array(np.arange(1.,mc+1.),np.float32)

b = np.array(np.arange(t,t+mc),np.float32)

c = np.array((1-psubd)**(np.arange(1.,mc+1.)),np.float32)

if (mf == mc):

d1mf = ((a/b)*c).sum()

d1mc = d1mf

d2mf = ((a[0:-1]/b[1:])*c[1:]).sum()

d2mc = d2mf

d3mf = ((a[0:-2]/b[2:])*c[2:]).sum()

d3mc = d3mf

d4mf = ((a[0:-3]/b[3:])*c[3:]).sum()

d4mc = d4mf

d5mf = ((a[0:-4]/b[4:])*c[4:]).sum()

d5mc = d5mf

else:

d1mf = ((a[0:-1]/b[0:-1])*c[0:-1]).sum()

d1mc = ((a/b)*c).sum()

d2mf = ((a[0:-2]/b[1:-1])*c[1:-1]).sum()

d2mc = ((a[0:-1]/b[1:])*c[1:]).sum()

d3mf = ((a[0:-3]/b[2:-1])*c[2:-1]).sum()

d3mc = ((a[0:-2]/b[2:])*c[2:]).sum()

d4mf = ((a[0:-4]/b[3:-1])*c[3:-1]).sum()

d4mc = ((a[0:-3]/b[3:])*c[3:]).sum()

d5mf = ((a[0:-5]/b[4:-1])*c[4:-1]).sum()

d5mc = ((a[0:-4]/b[4:])*c[4:]).sum()

# Using the quantities delta, d1, d2, d3, d4, d5, along with

# qexp, qobs, qref, and qinv, we are able to estimate the

# long term fitness value of employing each possible action

# These are denoted vexp, vobs, and vinv

vexpf = qexp * ( mf * deltamf + d1mf )

vexpc = qexp * ( mc * deltamc + d1mc )

vexp = mfw*vexpf + mcw*vexpc

vinvf = qinv * ( (mf-1) * deltamf + d2mf )

vinvc = qinv * ( (mc-1) * deltamc + d2mc )

vinv = mfw*vinvf + mcw*vinvc

# If we have at least 4 observations for estimating psubf,

# then we consider how the possibility of observation being

# a total failure affects the value of observing. We also

# do not let very young creatures consider this possibility

# as it may cause them to underestimate the value of observation

# early in life which can have disasterous results.

if ((roundsAlive > 5) & ((hmoves==OBSERVE).sum() > 3)):

#Find the probabilities of different numbers of failures (these sum to 1):

f0 = 1.-totpsubf; f1 = (1.-totpsubf)*totpsubf; f2 = (1-totpsubf)*(totpsubf**2); f3 = (totpsubf**3);

vobsf = qobs * ( f0*((mf-1) * deltamf + d2mf) +

f1*((mf-2) * deltamf + d3mf) +

f2*((mf-3) * deltamf + d4mf) +

f3*((mf-4) * deltamf + d5mf)

)

vobsc = qobs * ( f0*((mc-1) * deltamf + d2mc) +

f1*((mc-2) * deltamf + d3mc) +

f2*((mc-3) * deltamf + d4mc) +

f3*((mc-4) * deltamf + d5mc)

)

vobs = mfw*vobsf + mcw*vobsc

else:

vobsf = qobs * ( (mf-1) * deltamf + d2mf )

vobsc = qobs * ( (mc-1) * deltamc + d2mc )

vobs = mfw*vobsf + mcw*vobsc

#We then simply compare these values, and choose the action with the higher value.

#We intentionally order the potential moves so that the first one will be chosen in the event

#of a tie. The ordering choice is based on perceived risk aversion. Thus in the odd case where

#the expected value of exploiting is equal to that of observing, exploiting is the chosen action.

# pinvfail is part of a heuristic to discourage overenthusiastic innovation:

pinvfail = 0.95

pinvfail2 = 0.0

if obs_stale:

moves = [EXPLOIT, INNOVATE]

payoffs = np.asarray([vexp, vinv])

indx = (np.where(payoffs==payoffs.max()))[0]

if (moves[indx[0]] == EXPLOIT):

return (EXPLOIT, bestact)

elif (moves[indx[0]] == INNOVATE):

if (len(repertoire) < 2):

if (np.random.rand(1) < pinvfail2):

return (EXPLOIT, bestact)

else:

return (INNOVATE, )

else:

if (np.random.rand(1) < pinvfail):

return (EXPLOIT, bestact)

else:

return (INNOVATE, )

else:

pdb.set_trace() # this should never happen

else:

moves = [EXPLOIT, OBSERVE]

payoffs = np.asarray([vexp, vobs])

indx = (np.where(payoffs==payoffs.max()))[0]

if (moves[indx[0]] == EXPLOIT):

return (EXPLOIT, bestact)

elif (moves[indx[0]] == OBSERVE):

return (OBSERVE, )

else:

pdb.set_trace() # this should never happen

else:

# The basic premise of our strategy in the cumulative case is that

# there will typically be very few highly refined acts. In this case

# we say that refinement is saturated. When refinement is saturated

# critters should be exploiting as much as possible. In this case

# a simple strategy of observe (cleverly) and then only ever exploit

# (cleverly) may be unbeatable. Thus this strategy endevours to

# create a situation where there are only a very few highly refined

# acts, and then rely on it's ability to detect that indeed the act

# that they are exploiting is highly refined, and thus only exploit

# Though many strategies will certainly perform better than this

# before refinement has become saturated, we suspect that only an

# observe then exploit ever after type strategy will do well once

# refinment is saturated. Our hope is that prior to refine becoming

# saturated this strategy will have gained some small advantage over

# a naive observe exploit strategy, which will favour a higher

# frequency for this strategy in the count out even though the

# behaviour of this strategy and observe exploit should be nearly

# identical once refinement has become saturated.

# This is where we do all the decision making for the REFINE case:

refmoves = (hmoves == REFINE)

haveExploited = (hmoves == EXPLOIT).any()

haveRefined = refmoves.any()

haveInnovated = (hmoves == INNOVATE).any()

if ((~haveRefined) & haveInnovated):

# If the first act was learned through

# innovation, likely we are at the beginning

# of the simulation and this act should be refined

return (REFINE, bestact)

elif((~haveRefined) & (~haveExploited)):

# If nobs>1 the forager may decide to refine

# one of the observed acts, for simplicity we

# force the creature to exploit on that first

# round after an observe

return (EXPLOIT, bestact)

elif (~haveRefined):

# What follows are several very conservative

# hueristics for deciding prior to ever having

# refined whether it might be a good idea to

# refine.

if ((roundsAlive < 200) & ((alldistinctpayoffs.min()) < (alldistinctpayoffs.mean() * (5./7.)))):

# This is based on the notion that in the worst

# case scenario with rmax large, the increase

# from just one level of refinement is approximately

# 2.5 of the mean of the payoff distribution.

# If rmax is smaller, the ref bonus from a single

# refinement will be even larger.

# We use the minimum value ever perceived as a conservative upper

# bound on the current refinement level of all acts.

# If this leads to the conclusion the known acts

# are unrefined we refined them.

return (REFINE, bestact)

elif ((roundsAlive > 199) & ((alldistinctpayoffs.min()) < (alldistinctpayoffs.mean()) * (19./20.))):

# This is the same idea as above but now when the creature

# is older instead of asking if there is any refinement

# we ask are there at least 9 levels of refinement in

# the same worst case scenatio. Note that only about

# one percent of creatures live to this age.

return (REFINE, bestact)

elif ( (alldistinctpayoffs.min() < 16) & (qexp < 97) ):

# In the case where the mean of the payoff distribution

# is one, and rmax is large, then if the refinement level

# is less than 16 and the expected payoff from exploiting

# is less than 97, refining is worthwhile

return (REFINE, bestact)

elif ( (alldistinctpayoffs.min() < 34) & (qexp < 49) ):

# This is like the previous case

return (REFINE, bestact)

elif ( (alldistinctpayoffs.min() < 44) & (qexp < 25) ):

# This is also like the previous case

return (REFINE, bestact)

else:

return (EXPLOIT, bestact)

else: #haveRefined

# If you have decided to refine,

# just to be safe refine again.

if (refmoves.sum() < 2.):

return (REFINE, bestact)

else:

# Estimate the refinement bonus

# That is how much the payoff of

# the refined act will increase as

# a result of the refinement.

refindx = hmoves == REFINE

pay_post_ref = hpayoffs[refindx]

pay_pre_ref = hpayoffs[np.hstack((refindx[1:],np.asarray(False)))]

ref_increments = pay_post_ref - pay_pre_ref

refdecay = 0.95**(np.arange(len(ref_increments),0,-1))

refbo = (ref_increments * refdecay).mean()

if ((49.*refbo) > qexp):

return (REFINE, bestact)

else:

return (EXPLOIT, bestact)

def observe_who(exploiterData):

# Try to copy the creature with the highest payoff per round.

return sorted(exploiterData, key=lambda x: x[TOTAL_PAY] / (x[AGE]+1), reverse=True)
