# Supplementary material for: The refinement paradox and cumulative cultural evolution: Complex products of collective improvement favor conformist outcomes, blind copying, and hyper-credulity
Source: PLoS Comput Biol. 2024 Sep 26;20(9):e1012436. doi: 10.1371/journal.pcbi.1012436 (PMC11426424; doi:10.1371/journal.pcbi.1012436)
Supplement: Attachment — Submitted filename: Response_to_reviewers.docx [file pcbi.1012436.s002.docx]

**Response to referees**

We would like to thank the editor and the reviewers for all the helpful comments. We are particularly pleased to see a high number of suggestions for extensions of our work, which we take to suggest that reviewers found our approach fruitful in opening up further questions.

Below we address each remark point by point (the comments are in bold).

**Reviewer #1:**

**The central question in this article is of great interest to many of us researchers working on cultural and technological evolution: learning from others is natural, but the increase in knowledge and technological complexity over time is not. Why?

In the introduction, the article correctly identifies that the study of cultural complexity has been “hindered by the methodological challenge of cumulative culture, an inherently long-term process.” I concur that the artificial settings of controlled experiments (making paper airplanes, for example) have limited our understanding of cultural complexity. The lack of empirical studies is a significant barrier, as controlled experiments are insufficient to fully capture the complexity of empirical systems (see below). This, in my opinion, is a broader issue that affects the generality of lessons learned from theoretical studies of cultural evolution, including the present study. I'm concerned that the specific rules of the tournament may have biased the reported conclusions.

I must admit that I have struggled to interpret the findings of this study. This was most likely due to the tournament's somewhat abstract setting rather than a flaw in the paper methodology, which was well written and organized (although the analysis can be improved; see below). Aside from authoritative arguments, how can we be sure that the findings of these tournaments can be applied to real-world cultural evolution? For example, what is the rationale behind going through three different extensions? What actual system resembles a similarly staged selection process? An unambiguous method of demonstrating the validity of the chosen rules is urgently required. It would also be beneficial if the authors could motivate their research with a real-world scenario that is similar to the proposed tournament. This way, future expansions of the "tournament approach" will be built on a solid foundation.**

Response: We agree with the reviewer that it is always important to question theoretical constructs in this way, but in this case the three stages of the tournament did not attempt to simulate a real-world scenario. The three extensions (spatial, cumulative, model-biased) were our conditions of interest, and all three extensions have theoretical justification as to why the factors they explore might affect information ecology in cultural transmission contexts. The stages are heuristic devices that allowed us to look at the effects of each of the factors separately, and all three combined. We see this as a strength of this kind of modelling framework, as it allows us to investigate and combine these factors systematically rather than as the real world combines them. We have added a sentence in the main text (lines 266-267) to clarify this issue.

**The paper expands on a prior competition (“social learning strategies tournament”) that a subset of the authors organized in which 100 participants had to maximize their total payoff in a multiarmed bandit problem (Rendell et al. 2010). This approach was inspired by Robert Axelod's similar tournament in the 1980s, which investigated the evolution of cooperation. At each round, an individual must select one of three options/moves: "OBSERVE" to learn a behavior by observing another individual; "INNOVATE" to discover a new behavior on one's own; or "EXPLOIT" to obtain the current payoff associated with a known behavior. Individuals face a trade-off between receiving a payoff right away and learning a new and potentially better behavior that may result in payoffs later. The main conclusion of this tournament was that in an environment that is always changing, the more effective strategy is to periodically copy what others do instead of sampling the environment.

Moving on, determining the conditions under which cumulative cultural evolution can occur in this competitive environment is the primary contribution of the extended model. To achieve this, a fourth rule, 'REFINE', is added, which allows an individual to improve an existing behaviour. This new rule allows cumulative cultural evolution as other individuals can build on top of the improvements (“refinements”) made by other individuals. I found this rule to resemble more a kind of “parasitism” than a proper rule of complex cumulative culture. Many complex technologies require teamwork and cooperation among individuals, so one can imagine that the action of sharing information would be explicitly beneficial for both the demonstrator (the one executing “EXPLOIT” after doing some refinement actions) as well as the imitator (the one executing “OBSERVE”), as it happens in many open source software projects. How will the results of the tournament change if we make cooperation explicit?**

Response: We agree that our approach is just one, fairly simple, way of modelling social learning and refinement in this context. Given the complexity of the topic, we have chosen to start with what we think is a reasonable and readily comprehensible set of modelling assumptions. However, we agree that team effects and collaborative refinement are interesting questions and hope that future work will incorporate these extensions in a similar framework to ours. In terms of how this would change our results, we expect that any cooperative scenario where there is benefit also to the ‘demonstrator’ side will only make social learning more likely as it could benefit ostensive behaviours by the demonstrator and could be the start of the evolution of teaching behaviour – it could only really strengthen support for our results. Confirming this expectation is beyond the current study, however. We have added a section in the discussion focusing on interesting future questions (lines 418-422).

**Figure 1 is somewhat confusing to me because it implies some notion of progress, i.e., early forms of a technology yield higher pay-offs than more "finely tuned" recent versions. This contradicts the notion of an evolutionary process with no predetermined goal. On the other hand, I agree that such behavior may be valid for some (planned) technologies. Could you please provide the empirical data that informed the payoff curve for the Hammer case? Is there any empirical research showing that many technologies follow this curve? Or is it a heuristic assumption? To what extent do the results of the simulation depend on the specific shape of this curve?**

Response: We thank the reviewer for clarifying this. The diminishing returns curve is not intended to show a trajectory in evolutionary time, it is illustrating our heuristic assumptions about how the fitness benefits are associated with improvement. We think this is a reasonable assumption confirmed by previous work (Miu, E., Gulley, N., Laland, K. N., & Rendell, L. (2018). Innovation and cumulative culture through tweaks and leaps in online programming contests. Nature Communications, 9(1), 2321.). Diminishing returns curves have been used in previous work to model the relationship between time investment and improvement (Ugan, A., Bright, J., & Rogers, A. (2003). When is technology worth the trouble?. Journal of Archaeological Science, 30(10), 1315-1329. Frankenhuis, W. E., & Panchanathan, K. (2011). Balancing sampling and specialization: an adaptationist model of incremental development. Proceedings of the Royal Society B: Biological Sciences, 278(1724), 3558-3565.), along with other types of relationships (linear, exponential). In our model, the shape of the curve does not matter for our results as long as the payoffs reaped from refinement become higher than the base payoffs. In fact, the other curves (linear, exponential) imply a faster increase in payoff as refinement level increases, so comparatively our diminishing returns assumption is a conservative one. We have added clarification in lines 176-178.

**I agree that "conformity, blind imitation, and hyper-credulity" are intrinsically associated with cumulative culture. The title of the paper somehow points to "complexity [collective improvement in knowledge] favours conformity," but it seems to me the causality runs in the opposite direction, that is, "conformity hinders complexity." Complexity is not a mechanism but a systemic property that emerges from an evolutionary process based on multiple mechanisms, including conformity biases. I would appreciate it if the authors could clarify this point.**

Response: We absolutely agree that complexity is not a process, but an emergent property. We have made minor changes to the title to reflect this.

**A main result of the tournament is “finding that cumulative culture reduced cultural diversity” (line 283). Our most recent empirical study (which the authors did not appear to notice) supports this conclusion. In it, we measured how excessive imitation greatly decreased the cultural diversity of three large-scale real-world systems (see Duran-Nebreda, S., O’Brien, M. J., Bentley, R. A., and Valverde, S. (2022) “Dilution of expertise in the rise and fall of collective innovation,” Humanities & Social Sciences Communications, 9:365). Cumulative culture (along with complexity and diversity) can collapse due to the effects of “over-imitation” (line 48). We developed a theory that makes testable predictions about the loss of cultural diversity in real systems. For a robust (and system-independent) assessment of cultural trends, we have used three different measures of cultural diversity, including Kolmogorov complexity, which anticipates cultural collapses.

It would be beneficial to discuss (at least qualitatively) these two approaches, which appear to represent different hypotheses about the decline in cultural diversity. While the tournament attributes complexity to external drivers, such as "spatial and environmental variation in payoffs, combined with societal differences in values and utility functions, generate and preserve cultural diversity" (lines 287-290), our empirical analysis suggests an endogenous origin. This distinction appears to be the result of the tournament assuming that cultural evolution generates complexity primarily through competition rather than cooperation. The readers of the paper would greatly benefit from an explicit comment on this point in order to provide a fair representation of current approaches linking conformity with the loss of cultural diversity.**

Response: Although we have discussed mainly external drivers of diversity, we agree that endogenous factors are crucial for explaining such losses of diversity, and gladly welcome the reviewer’s suggested citation, which is both very valuable and fits well with the results presented here. We have now added a section in the discussion where we discuss our results in light of this paper (lines 396-403, lines 409-410).

**For example, what kind of functional curve corresponds to the evolutionary trajectories of cultural diversity (Figure 3)? They appear to be exponential, but we need to run a fitting test. It would be great if we could explain the fitting curve in terms of tournament parameters and measurements. In addition, some panels in Figure S4 show what appears to be a punctuated equilibria, with a sharp decay at around t = 15 in the curves of "mean knowledge amount" and "mean knowledge evenness." What is the origin of this shift? Many evolutionary algorithms display similar behaviors. In the context of cultural evolution, I am curious if this drop can be explained as a trade-off between conformity bias and the strength of selection (as suggested in Vidiella et al., 2022, "A cultural evolutionary theory that explains both gradual and punctuated change," J. *R. Soc. Interface 19: 202220570).***

Response: We thank the reviewer for pointing this out. We explain the shape of the curves as a result of individuals knowing and using the same acts, driven by the use of social learning in a refined world. When refined acts are higher payoff than unrefined acts, and agents can learn from each other, we observe this conformist outcome although our tournament does not implement conformist bias. Hence, while the reviewer’s hypothesis may be generally plausible, it is not the explanation here. As the reviewer noted, this decrease in diversity happens very quickly. We have not attempted to fit a function to this curve and explain it as a result of other tournament parameters because we think the explanation above is both accurate and sufficient.

The dip in figure S4 is explained by the fact that, in order to accurately measure the frequency of each strategy and therefore measure their score, the simulations contained no mutation in the last quarter of the simulation (timesteps 15-20). With mutation turned off, the conformist outcome mentioned above becomes even more evident. The fact that the shape of the curve is a result of a modification in the procedure we used to run the simulations provides further justification for avoiding curve fitting. However, we are grateful to the reviewer for pointing out this potential source of confusion, and have now clarified this point in the article.

**Additional metrics could help to strengthen the arguments presented in the paper. The use of entropy-based measures of diversity has the disadvantage of producing similar values of evenness for very different distributions. To better understand how the system is behaving, we need to characterize the empirical probability distribution, p_i, or the proportion of agents using behavior i, which is used to compute the evenness (as described in lines 598-602). Please plot this distribution (at various points in time) in a figure so that we can better understand how the behavior/traits are distributed in the population. If the distribution has a long tail, such as p_i ~ i^{-\alpha}, the exponent is a more accurate measure of diversity and can be linked to evolutionary mechanisms, such as conformity (Duran-Nebreda et al., 2022). In addition, the plots in panels F and H in Figure 3 suggest a heterogeneous distribution of the maximum and minimum knowledge persistence.**

Response: We thank the reviewer for this comment and agree that more transparency in our diversity measures is beneficial. We have now added a new figure in our Supplementary Information (Fig. S6) that plots the proportion of agents who know and use each behaviour (p_i) at different time points, alongside the evenness measure at the same timepoints, extracted from one simulation in the cumulative extension in Stage II. We hope this illustrates that the p_i distributions change over time, but very quickly become very skewed – in this example for the great majority of the simulation the population knows and uses only one act.

**Finally, Figure S8 is very interesting, as it suggests a possible classification of the strategies based on the distance between the corresponding probability distributions of "REFINE moves." For example, "Grandma," "apc," "ShamelessExplorer," and "LifeLongLearner" have similar bimodal distributions and could be classified as a single group; however, "followTheMeans," "mayFlower," and "fiveYearPlan" have a similar long-tail distribution (power-law?) indicating a different class of behavior. The clustering of strategies could be summarized in a cladogram and discussed in the main text, allowing the reader to visually understand the breadth of strategies proposed.**

Response: We thank the reviewer for the interest in this figure. We also thought such a classification was interesting, and added this figure in the supplementary material to spark interest in future research, although further exploration is beyond the scope of the current study. We have provided the full code and data used for this study, and hope that scholars interested in additional analyses could pick this up and build on it. The current study is only scratching the surface of the potential of this framework, but we think the paper is already too dense to allow for the inclusion of further analyses beyond what we included here.

**Reviewer #2:**

**The manuscript investigates the phenomenon of cumulative culture. Through an exhaustive and well designed experiment conducted via a computer tournament, the authors delve into the refinement paradox, where refining behavior yields higher payoffs but does not generally pay off. Their findings shed light on the intricate dynamics underlying the evolution of knowledge and technology, revealing that while sophisticated strategies initially benefit individuals, complex culture ultimately fosters conformity and blind imitation. I really like this research and the conclusions. Nevertheless, I suggest the authors consider the optional subsequent comments:

1. Order of Concepts: The manuscript's structure is commendable, providing a comprehensive exploration of complex concepts. However, there are instances (for example, melee) where concepts are introduced before their definitions, which may momentarily confuse readers. Integrating brief definitions when introducing new concepts would further enhance the manuscript's clarity and accessibility.**

Response: We thank the reviewer for pointing out this lack of clarity. In response to this and to comments from Reviewer 3, we have reorganised the section that introduces the tournament and have added considerable detail. We hope that this restructuring solves some of the issues pointed out here and introduces the concepts more thoroughly.

**2. Number of Moves: The experimental setup with four moves is intriguing, yet the rationale behind this choice is not explicitly discussed. Considering the significance of this decision, providing insight into why four moves were chosen and discussing the potential influence of this parameter would enrich the manuscript's depth and completeness.**

Response: We chose four moves because we aimed to model the simplest framework that could assess the trade-offs between the four relevant factors: social learning, individual learning, refining, and exploiting. The current framework is building on previous work that investigated the relative benefits of social and individual learning. EXPLOIT was deemed necessary to asses the trade-off between learning and using the information learned. In this study we were interested in how refining, a specific type of learning, interacts with these factors. Therefore, to maintain feasibility, we merely added an extra move, REFINE. The aim of this paper was to describe how adding this fourth move changed the results of our previous work that only implemented three moves. Of course, we can imagine other types of learning might be relevant for cultural adaptation, but these are beyond the scope of the current study. We added clarification in lines 124-127.

**3. Missing Reference: While the manuscript correctly references Render et al. 2010, there appears to be a missing reference with Render et al. 2011.**

Response: Thank you for pointing this out, now fixed in line 84.

**4. Preregistration: Preregistration of experiments enhances research transparency and reproducibility. I don't know if it was preregistered, although it does not affect my positive opinion about this research.**

Response: We thank the reviewer for raising this pertinent point. We agree that research transparency and reproducibility and important and necessary considerations, that we are also committed to. Our tournament was run in 2012, before preregistration was a widespread norm, but this nonetheless did not preclude us from sharing our code and our data on OSF, for anyone interested in reproducing our results.

**Reviewer #3:**

**The paper addresses the emergence and rarity of cumulative culture. I believe
it's a great contribution to the field of cultural evolution. The authors
present a sophisticated and well-designed computer tournament to explore the
conditions under which cumulative culture -- characterized by increasing
complexity and diversity of knowledge and technology -- can evolve. This study
is not only innovative but also highly insightful, shedding light on the
refinement paradox and the intricate balance between knowledge improvement and
the benefits of simple imitation. The clarity of presentation is commendable,
although the complexity of the simulation experiment might be daunting for
readers not well-versed in such methodologies. Nevertheless, the findings are
profoundly exciting and add a critical layer to our understanding of cultural
evolution, particularly the nuanced dynamics that govern the rarity and
emergence of cumulative culture in nature. Below are some minor comments with
which I hope the authors can further improve their study.

* Comments on Clarity and Structure
- Make it clearer from the start how the different moves (INNOVATE, OBSERVE,
EXPLOIT) should be categorized. An important distinction is obtaining payoff
versus learning about payoff. Another is the accurate learning or error-prone
estimation of payoff. Explicitly defining these categories early on would help
the reader understand the roles and interactions of each move, improving the
overall flow and comprehension of the text.**

Response: We thank the reviewer for pointing our this lack of clarity. In response to this comment and the following comment, we have now restructured the initial description of the tournament, adding more detail. We now mention specifically which moves are learning moves, and which are exploit (lines 123-125), mention specifically the trade-off between learning and exploiting (lines 208-210), and mention specifically which are error-prone and which are not (lines 213-216).

**- For the paragraphs describing the different moves, consider restructuring this
section to better prepare the reader for the various components of the model.
The current presentation is somewhat verbose and lengthy, making it
challenging to grasp how all the elements connect. The materials and methods
section is much clearer than the earlier explanations. Therefore, rearranging
the paper to present the more detailed descriptions before discussing the
results would provide readers with crucial details upfront and enhance their
understanding of the study.**

Response: We thank the reviewer for this comment. We have now restructured the section that introduces the tournament and moved much of the decription of the four moves from the Materials and Methods section to the main text (lines 134-216). We hope this clarifies the structure of the model.

**- At the introduction of the INNOVATE move, it is unclear why this involves
accurate information about payoff. Similarly, it is unclear why the payoff is
estimated without error when observing. Clarifying these points would help in
understanding the rationale behind these assumptions and their implications
for the simulation results. This might however be resolved when rearranging
certain parts.**

Response: While in practice there will be some level of error associated with asocial learning (e.g. in estimates of the payoff to their own behavior), it is a reasonable assumption that any such error will generally be low relative to the error in estimates of the payoff that another individual receives associated with social learning. Here, the error associated with social learning can reasonably be construed as representing the difference in error associated with the two forms of learning. That social learning is generally more error prone than asocial learning is a common assumption in the literature (e.g. Boyd & Richerson 1985). We have clarified this in the text (lines 213-216).
 **- It would be helpful to clarify from the start that this is a real-world
tournament. Due to the simulation context, I initially thought the designers
of the entries were also simulated. Adding a brief explanation about the
real-world nature of the tournament and the involvement of actual participants
would prevent this confusion. Additionally, providing more information about
the backgrounds of the designers of the entries could offer valuable context
and enhance the reader's appreciation of the tournament's scope and diversity.**

Response: We agree that the complexity of the framework was not clearly described in the main text. We have now added a section clarifying that the entrants were people rather than simulations, and describe the backgrounds of the entrants who did share this with us (lines 83-90).

*** Comments on Methodology and Analysis

- While using Pielou's Evenness measure is an apt choice, it would be insightful
to compare it with alternative measures of evenness. This comparison would
provide a more complete understanding of the distribution of behaviors. See
Chao's work on Hill Numbers and Evenness for a more comprehensive presentation
(**[**https://doi.org/10.1002/ecy.2852**](https://doi.org/10.1002/ecy.2852)**). Currently, to appreciate the point that
diversity decreases and, simultaneously, the remaining traits are distributed
less evenly, we need both Figure 3A and 3C. The evenness profiles proposed by
Chao would allow the authors to present this is a single graph.**

Response: We thank the reviewer for this suggestion and for pointing out new developments in this area. We have chosen the diversity measures used in this paper because they are both appropriate and accessible measures that many readers would be familiar with. In our reading of the cited paper, we understand the main contribution of Chao’s evenness to be that it allows to incorporate ecological function data and phylogenetic relationships between species, neither of which we have available for our data.

However, we take the reviewer’s point that it would be useful to add more detail on our diversity measures. To this end, in response to this comment and to comments from reviewer 1, we have now added a new figure in our Supplementary Information (Fig. S6) that plots the proportion of agents who know and use each behaviour (p_i) at different time points, alongside the evenness measure at the same timepoints, extracted from one simulation in the cumulative extension in Stage II. We hope this illustrates that the p_i distributions change over time, but very quickly become very skewed – in this example for the great majority of the simulation the population knows and uses only one act.

**- The authors show that social learning in general has a negative effect on the
diversity of behaviors, and that's also the case in the cumulative setting
with the refine move. It seems, however, that even without refine there is
still considerable decline in diversity as social learning increases. Would a
more comprehensive ablation study or model comparison help single out the
impact of different moves more precisely on observed diversity changes? I did
not find an explanation for this.**

Response: We agree with the reviewer that even without refinement, social learning results in reduced diversity, as the population converges on the more useful behaviours. This dynamic was explored in our previous work (Rendell 2010, 2011). Here we showed that the possibility of refinement and social learning in a refined world results in even more dramatic reductions in diversity (in Fig. 3a-b the agents use approx. 1-2 behaviours). We do not see these two results as arising from different mechanisms – in both cases social learning helps agents land on beneficial behaviours, but in the refinement case what is beneficial is more homogenous. With this in mind, we think that all moves are necessary for this dynamic to emerge. This dynamic was the focus of our investigation here, but we think part of the value of our framework is the richness of analyses it allows for, richness that we do not hope to be able to fully explore in this paper. We hope that future work can build on this and similar frameworks and address such further questions.

*** Contextual Considerations

- In the simulation context, payoff benefits only the individual. In real-world
social contexts, payoffs can benefit more people (e.g., kin, friends). What if
the inner circle of an individual is enlarged to cover kin? This would make
the greater good on a smaller scale a more direct objective of an individual.
Exploring how social relationships and communal benefits influence payoff
dynamics could add an important dimension to the study's implications.**

Response: We thank the reviewer for suggesting this very pertinent point. We agree that a framework like this could and should be extended to capture cooperative dynamics. Reviewer 1 has suggested a similar extension that would capture collaboration and team effects. While we think these points are both interesting and necessary future steps, they go beyond the scope of this study. However, we think that our model here provides a foundation on which these compelling questions can be explored, and we have provided our code and our data with the hope that interested researchers could attempt such these extensions. We have added a section in the discussion focussing on interesting future questions (lines 419-422).

***- The paper focuses on culture defined by material reflections such as tools and
technology. Can the authors also reflect on the emergence of other aspects of
culture that develop cumulatively but without clear payoffs, such as
storytelling, music, etc.? Including a discussion on non-material cultural
elements would broaden the scope of the study and highlight the diverse forms
of cumulative culture.***

Response: We thank the reviewer for this great point on what is currently an active theoretical debate in the literature. Here we have focused on tools and technology as the archetypal case of cumulative cultural evolution because they capture fairly transparently the adaptive nature of human-like culture. Humans have been able to improve technology and knowledge about the world in order to better their lives, with direct effects on survival rates. How less functional domains, like music or aesthetic domains, affect survival is less obvious, and might need a careful distinction between biological and cultural fitness. To be clear, we are not claiming that non-technological domains do not have adaptive value, but the shape of that relationship between refinement and adaptive value is less well understood, and likely to be frequency-dependent in a way that would require a rethinking of our simulation structure that is beyond our current scope. We have added a section in the discussion that grapples with these issues (lines 414-418).

*** Technical Comments

- The code is in Python 2.7, which is no longer supported. This makes it
challenging to run and test the code. Is Python 2.7 necessary, or could the
code be updated to more modern standards? Updating the code to a current
version of Python would enhance its accessibility and usability for other researchers.**

Response: We thank the reviewer for this point, which has made us reflect on the timeline of a project like this and its implications for code maintenance. We agree that code accessibility should be a main priority, and we have converted the main simulation package and two simple demonstration strategies to Python 3 for the convenience of interested parties. Therse are also available on the OSF repository. Most of the entries, which have not been written by us but by our entrants, are still Python 2.7 compatible. It did not seem appropriate to rewrite what is, in the case of this study, data, but we find that Python 2.7 is still easily available for download (e.g. WinPython 2.7 <https://sourceforge.net/projects/winpython/files/WinPython_2.7/> or as an Anaconda environment <https://docs.conda.io/projects/conda/en/4.6.1/user-guide/tasks/manage-python.html>) and think that, in this case at least, using an older version might be the best solution.

**- Provide an example in the section describing entries as computer code
functions. This would greatly aid in understanding how these functions
operate. An example would illustrate the practical implementation of the model
and clarify the decision-making process of the agents.**

Response: We agree that example entries would benefit our presentation – we have now added the winning entry, farSightPolymorph, as an example in the Supplementary Information, and have added a number of entries from entrants who agreed to share theirs to our OSF repository.

**- Add information about cultural diversity quantification to caption of figure 3A
and B. This detail is crucial for understanding the results.**

Response: Thank you for asking for pointing out this lack of clarity, we have now added more detail in the caption for figure 3.
